# The Current Week's Proceedings

**Published:** 1915-10-02

**Authors:** 


### PARLIAMENT AND THE PROFESSION.

### THE CURRENT WEEK'S PROCEEDINGS.

### Hospitals and the Spirit Tax.

In his Budget speech Mr. McKenna made no allusion to the promised concessions to hospitals in the matter of duty-free spirit, and it does not appear probable that any provisions for the concessions will be contained in the Finance Bill. There is, in fact, good reason to believe that the Government proposals will not be introduced until the earlier part of next year. For this delay the hospital world has to thank the British Medical Association!

### Increased Duty on Patent Medicines.

The increase in the tax on secret and proprietary medicines, for which provision was made in one of the resolutions passed on Budget night, is not likely to have any material influence on the demand for these remedies. Mr. McKenna's estimate that by doubling the tax the extra revenue from this source would be a quarter of a million sterling allows for a falling-off of about 10 per cent., but it is doubtful whether the decline will be even as much as this. This is one of the extra taxes that might well be retained permanently; it is fitting that the public should be made to pay for its folly.

### Income Tax on Doctors' Book Debts.

A matter of direct interest to medical men was referred to by Sir Hildred Carlile when he asked the Chancellor of the Exchequer whether surveyors of taxes are directed, when making an assessment for income tax, to add to the proved incomes of the medical profession one-third, or, indeed, any part of the value of any book debts which may be standing in their books, and which they may never succeed in recovering. Mr. McKenna's reply was that no instructions of the nature suggested had been issued. In the case of medical men, as in other cases, any sum for book debts which has been included for income-tax purposes in the profits of any year is, of course, excluded from the profits of the year in which the debts are paid. Should the debts prove to be irrecoverable they would be allowed as a deduction from the profits of the year in which they were subsequently written off.

### Gallant Deeds of Army Doctors.

Replying to a question asked by Mr. T. M. Healy, Mr. Tennant stated that the mere fact of being wounded whilst attending the soldiers under fire does not entitle a medical officer to special recognition for bravery, but these officers receive full recognition for valour shown, their cases being considered on the same lines as those of other officers. Any exceptional circumstances would be given their proper weight. Lists of awards for gallantry by members of the R.A.M.C., which have from time to time been published in The Hospital, clearly show that the brave deeds of army surgeons are not overlooked.

### Mental Disturbances and the War.

One of the saddest problems of the war was referred to by Mr. Touche when he asked the Under-Secretary of State for War what hospital provision, apart from lunacy control and association, with its industrial and social prejudice, had been provided where uncertifiable cases of nerve disturbance and loss of mental balance amongst the rank and file, requiring special and continued treatment, could be placed under special medical care. Mr. Tennant's reply was to the effect that cases of the kind mentioned are treated in the neurological sections of twenty-three military hospitals in the United Kingdom. Accommodation for these cases is also afforded by the Springfield House Hospital, Wandsworth, and the Red Cross Military Hospital, Maghull, near Liverpool. Unfortunately, the accommodation for cases of the kind in question will have to be greatly extended in the near future. With regard to the more serious mental cases the disposal of these has been receiving consideration, and has caused the authorities much anxiety. Owing to the increasing numbers of incurable cases, Mr. Tennant says it will not be possible to continue indefinitely the system of retaining under the control of the Secretary of State cases of general paralysis, chronic epilepsy, and chronic insanity which have had previous asylum treatment.

### Trench Fever.

The Under-Secretary of State for War has given his assurance that careful records of all cases of trench fever are being preserved, and has repudiated the suggestion that men suffering from typhoid will be diagnosed as suffering from trench fever. The Army medical authorities will not diagnose cases as trench fever unless the results of the bacteriological examination justify such a diagnosis. The subject of trench fever is being specially investigated at the present time.

### Refusing to be Vaccinated.

A curious report was referred to in the House the other day by Mr. Chancellor. He asked the Under-Secretary of State for War whether he was aware that a certain private in a Territorial regiment was awarded twenty-one days' confinement for refusing to be vaccinated; and, if so, whether this punishment for the exercise of a legal right was authorised. Mr. Tennant, in his reply, stated that the report was incorrect. The soldier's offence for which he was awarded twenty-one days' confinement to barracks was not refusing to be vaccinated, but refusing to obey a military order to parade and inciting other men to refuse to parade or to be vaccinated. This is quite a different matter, but those people who are so terribly shocked at the idea of punishment for refusing to be vaccinated should read the note in The Hospital of last week (p. 539) concerning the French system of dealing with such cases.

### An Irish Grievance.

The action of the Irish Insurance Commissioners in setting aside the present certification arrangements for insured persons requiring benefit in Ireland is said to threaten at least 200 doctors with loss of office and of the salary agreed on. Mr. J. P. Farrell, in drawing the attention of the House of Commons to the matter, asked whether it was proposed to proceed with the new arrangements in violation of obligations incurred by doctors. The reply was that the part-time appointments were made on a temporary basis pending the settlement of a permanent scheme for certification, and were terminable by one month's notice on either side. This fact was clearly indicated by the Irish Insurance Commissioners to all candidates for such appointments. The matter has not been definitely settled, however, and the Insurance Commissioners will receive a deputation from the medical men affected.

